# Duodenal Web in an Elderly Patient with Abdominal Discomfort and Vomiting: A Rare Case

**DOI:** 10.34172/aim.2023.69

**Published:** 2023-08-01

**Authors:** Mehdi Alimadadi, Reza Ghanbari, Mohanna Yousefi, Somayeh Sadani

**Affiliations:** ^1^Clinical Research Development Unit (CRDU), Sayad Shirazi Hospital, Golestan University of Medical Sciences, Gorgan, Iran; ^2^Gene Therapy Research Center, Digestive Diseases Research Institute, Tehran University of Medical Sciences, Tehran, Iran

**Keywords:** Delayed presentation, Duodenal obstruction, Duodenal web, Surgical excision

## Abstract

Duodenal web is complete or incomplete obstruction of the duodenum due to a membranous web or intraluminal diverticulum. This abnormality is one of the main causes of intestinal obstruction in children. The symptoms of this disease may rarely appear in older age and cause gastric outlet obstruction in adults. In the present paper, we report a 69-year-old male patient with heartburn, abdominal discomfort, frequent non-bilious, non-bloody vomiting for the past 6 months. Furthermore, the patient had experienced a weight loss of 12 kg during this period. He had been taking aspirin daily for years due to his ischemic heart disease. After performing contrast-enhanced CT imaging, esophagogastroduodenoscopy and barium meal examination, the patient was diagnosed to suffer from duodenal web. Since surgery is currently the mainstay of treatment in the management of this disease, the patient finally underwent a gastrojejunostomy.

## Introduction

 A duodenal web is a rare congenital defect that occurs as a result of incomplete duodenum recanalization between the 6th and 8th weeks of gestation.^1^ It has been shown that duodenal web and duodenal atresia can cause intestinal obstruction in children, and their incidence is 1 per 10 000 to 40 000 live births.^2^ The web itself consists of a thin layer of mucosa and submucosa without muscularis, while duodenal atresia is more common and includes muscularis propria.^3^ This abnormality appears mainly in infancy, but it has also been reported in adults. Many of these patients usually suffer from long-term complications before being diagnosed or starting the treatment process.^1,4^

 Adult patients with duodenal web usually present with manifestations of gastric outlet obstruction, including symptoms of abdominal and epigastric pain, postprandial nausea, vomiting and weight loss.^5,6^ In adults, it is usually diagnosed with a delay, due to non-specific symptoms and difficult visualization of the web on endoscopy, and this delayed diagnosis usually leads to significant complications for the patient.^7^ Differentiating whether the duodenal web in adults is congenital or acquired (e.g. as a result of long-term non-steroidal anti-inflammatory drugs use, or secondary to pancreatitis, ulceration, obstruction or abdominal surgery) could be difficult.^1,8^

 Here, we report a case of delayed presentation of second part duodenal web diagnosed in a 69-year old male patient with abdominal discomfort, vomiting and weight loss.

## Case Report

 A 69-year-old male patient referred to us with complaints of exacerbation of heartburn, abdominal discomfort, frequent non-bloody, non-bilious vomiting and abnormal sensation of food remaining lodged in the throat or chest since six months ago. The patient also experienced a 12-kg weight loss during this period. He was a smoker and also addicted to opium and his frequency of defecation was once every other day. He had a history of heartburn and dyspepsia since he was 30 years old, and took proton pump inhibitors occasionally, but over the past 6 months, he woke up several times during the night due to the severity of his symptoms. The previous medical history of the patient was ischemic heart disease and he was taking aspirin daily. The patient was in good general condition and his vital signs were stable; no abnormalities were found on physical examination.

**Figure 1:**
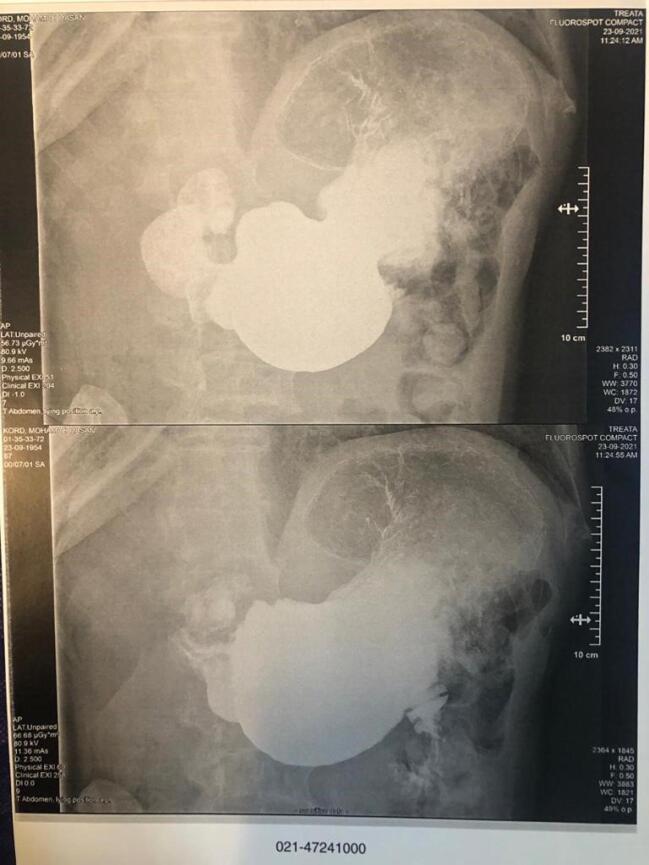
Barium Meal Examination. Accumulation of barium inside the distended stomach and slow passage of barium through the duodenum

**Figure 2:**
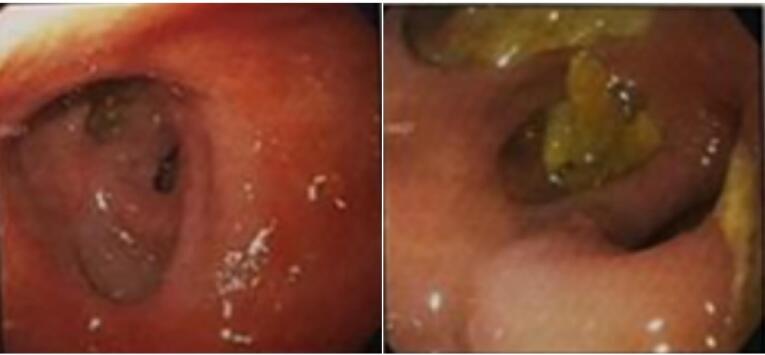
Esophagogastroduodenoscopy on the patient for definitive diagnosis. (left) A deformity and diverticulum in the duodenal bulb. (right) A food debris accumulation and diverticulum in the prepyloric area.

**Figure 3:**
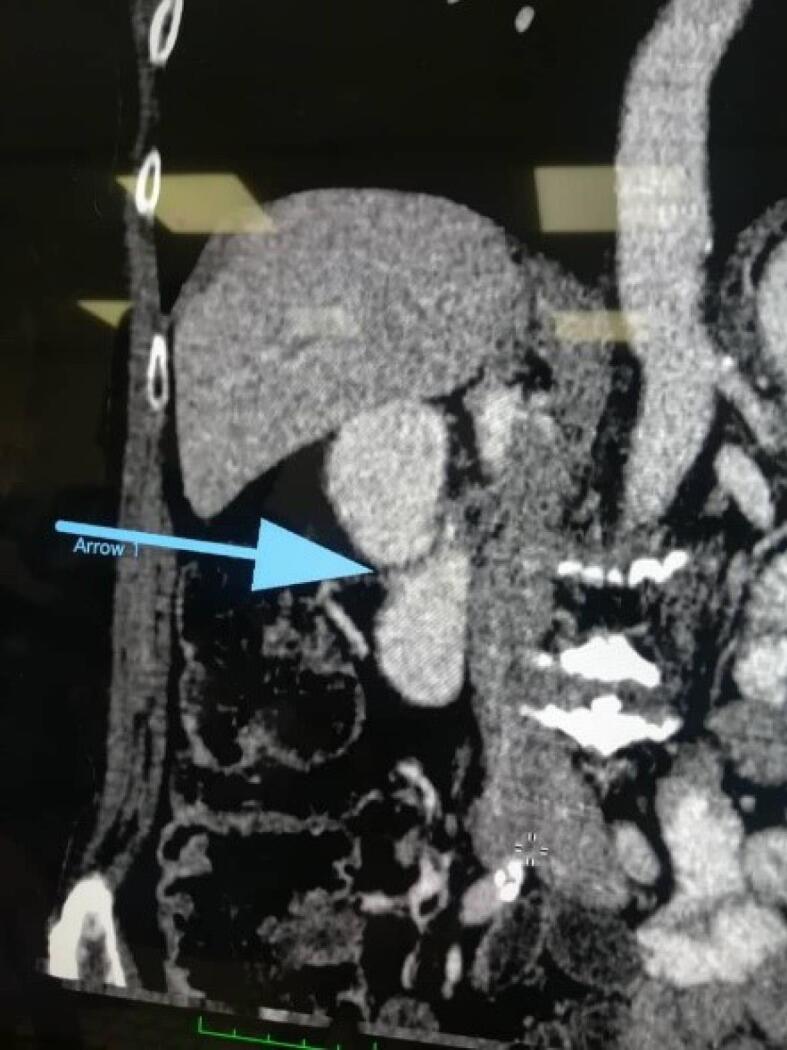
Linear-shaped Filling Defects in the Second Part of the Duodenum on Contrast-Enhanced CT

 Contrast-enhanced computed tomography (CT), esophagogastroduodenoscopy and barium meal examination were performed on the patient to yield a definitive diagnosis. As shown in Figure 1, the result of the barium meal examination was normal. After barium swallowing, the esophagus showed normal peristalsis and the mucosal pattern was normal. The barium sulfate suspension passed easily through the pylorus. Esophagogastroduodenoscopy revealed a diverticulum in the prepyloric and duodenal bulb area, such that the scope could not pass through the D2 (Figure 2). Furthermore, according to the oral and intravenous contrast-enhanced CT, there was a linear-shaped filling defect in the second part of the duodenum, suggesting a web or congenital structure (Figure 3). No intra-abdominal free fluid or free air was found. Also, the liver, gallbladder, and pancreas were normal.

 Finally, to start the treatment process, the patient was referred for surgical exploration. Considering the patient’s age, preoperative echocardiography was undertaken, and the ejection fraction was 55% without any abnormality. After initial assessment and hemodynamic stabilization, the patient underwent laparotomy and gastrojejunostomy. On the third postoperative day, the patient was allowed oral intake, starting with clear liquids, which were well tolerated. After the operation, the patient had no problem with defecation and was also able to take a full mixed diet by the seventh postoperative day. Finally, the patient was discharged in good general condition with no complaints of symptoms.

## Discussion

 Duodenal web is a membrane-like structure with the ability to partially or completely obstruct the duodenum. Although most of the webs are formed in the pre- and post-ampullary regions, they have been also reported throughout the third and fourth parts of the duodenum.^3^ Duodenal webs may be complete, in which case it closely resemble duodenal atresia and can be fatal if not treated in early infancy, while incomplete webs usually manifest as partial obstruction with a wide range of symptoms.^3^ Webs mainly appear in infancy, but there have been reports in adulthood as well. Detection of this abnormality in adulthood is challenging, both because its symptoms are non-specific and it may be difficult to visualize on endoscopy; due to the delayed diagnosis of this defect, the final management and treatment of these patients is often postponed.^9,10^ Our case was a 69-year-old male patient with symptoms of abdominal discomfort and vomiting, who was found to be suffering from duodenal web in the second portion of the duodenum (D2).

 According to the patients’ clinical and radiological findings, it is possible to find out the presence of duodenal web in patients.^11^ Studies conducted on identification of patients with duodenal web have shown that endoscopy, contrast-enhanced CT imaging, and barium meal examination are used for accurate diagnosis of gastrointestinal obstruction.^12^ An upper gastrointestinal contrast study could be the method of choice to establish the diagnosis of this anomaly, especially for patients with older age and also with partial obstruction, such as the case evaluated in the present study. In addition, flexible endoscopy could also reveal the dilatation of the duodenum and the protrusion of the web into the lumen.^13,14^ In this study, for our case, a barium meal examination was performed first, and the result was normal. Subsequently, contrast enhanced CT with oral and intravenous contrast was performed for our case, which revealed linear-shaped filling defects in the second part of the duodenum, indicating a web or congenital structure. Finally, esophagogastroduodenoscopy was performed for the patient, the findings of which indicated a diverticulum in the prepyloric and duodenal bulb; in addition, during this process, the scope was not able to pass through the second part of the duodenum (D2) due to obstruction.

 Most patients with duodenal web also have peptic ulcer disease, which usually resolves after successful management of the duodenal web.^15^ Adults with duodenal web often present with symptoms of abdominal and epigastric pain, nausea, vomiting, abdominal distension, weight loss and gastric outlet obstruction. Due to gastric outlet obstruction, heartburn and acid reflux symptoms are common in these patients.^5,6,15,16^ The patient in our study also suffered from symptoms of heartburn, abdominal discomfort, vomiting and abnormal sensation of food remaining lodged.

 There is a strong association between congenital duodenal web and other congenital anomalies such as trisomy 21, congenital heart disease, and developmental delay.^4,17^ There is not much information available about the prevalence of duodenal web in adults, because most of them are misdiagnosed.^1^ In an autopsy series conducted by Ravitch, the incidence of this disease in adults was estimated at 3 in 20 000.^18^ In addition, in a retrospective study conducted by Ladd et al in a single center over a period of 16 years, only 16 patients with congenital duodenal obstruction were identified.^1^

 The duodenal web in adults may appear as a secondary lesion in pancreatitis, ulceration, abdominal surgeries, obstruction and chronic use of non-steroidal anti-inflammatory drugs (NSAIDs).^8^ It has been shown that the most probable cause of duodenal web development in adulthood is long-term use of NSAIDs. The exact mechanism of the relationship between chronic NSAID use and the formation of duodenal web has not yet been properly identified, but these drugs can cause intestinal ulceration and it is assumed that the granulation tissue of the wound matures into a scar; ultimately, scar contraction can be the foundation of web formation.^19^ The case presented in this study had no history of any of the above-mentioned diseases, nor did he take NSAIDs. Our case only used aspirin daily due to his history of ischemic heart disease.

 The mainstay of treatment in these patients is surgical management.^14^ In managing these patients, it is important to note that not all of these patients need surgery, but in patients with significant obstruction, surgery could be the best management. In most cases, the duodenal web is completely removed or dilated by surgery or endoscopy. In our case, we performed a successful gastrojejunostomy. After surgery, he tolerated food intake and had defecation. He was discharged in good condition with no complaints about his symptoms.

 In conclusion, abnormal clinical symptoms in infants are usually considered in terms of the presence of duodenal web, but in adults, the differential diagnosis of duodenal web is rarely considered, which can be one of the reasons for its delayed diagnosis. Delayed diagnosis of this abnormality in adults is common, which can cause long-term complications such as malnutrition and weight loss.^7^ It is suggested that on radiographic and endoscopic evaluation of patients at any age, the duodenal web should also be considered in order to prevent the long-term suffering of patients.
